# Landscape characteristics influence ranging behavior of Asian elephants at the human-wildlands interface in Myanmar

**DOI:** 10.1186/s40462-022-00304-x

**Published:** 2022-02-05

**Authors:** A. N. Chan, G. Wittemyer, J. McEvoy, A. C. Williams, N. Cox, P. Soe, M. Grindley, N. M. Shwe, A. M. Chit, Z. M. Oo, P. Leimgruber

**Affiliations:** 1 Conservation Ecology Center, Smithsonian National Zoo and Conservation Biology Institute, Front Royal, VA 22630 USA; 2 Department of Fish, Wildlife, and Conservation Biology, Colorado State University, Fort Collins, CO 80523 USA; 3 WWF-Myanmar, Yangon, Myanmar; 4 Fauna and Flora International, Yangon, Myanmar; 5 Ministry of Natural Resources and Environmental Conservation, Myanma Timber Enterprise, Alone, Yangon, Myanmar

**Keywords:** Asian elephant, Animal movement, GPS tracking, Home range, Ranging behavior, Landscape ecology, Myanmar

## Abstract

**Context:**

Asian elephant numbers are declining across much of their range driven largely by serious threats from land use change resulting in habitat loss and fragmentation. Myanmar, holding critical range for the species, is undergoing major developments due to recent sociopolitical changes. To effectively manage and conserve the remaining populations of endangered elephants in the country, it is crucial to understand their ranging behavior.

**Objectives:**

Our objectives were to (1) estimate the sizes of dry, wet, and annual ranges of wild elephants in Myanmar; and quantify the relationship between dry season (the period when human-elephant interactions are the most likely to occur) range size and configurations of agriculture and natural vegetation within the range, and (2) evaluate how percentage of agriculture within dry core range (50% AKDE range) of elephants relates to their daily distance traveled.

**Methods:**

We used autocorrelated kernel density estimator (AKDE) based on a continuous-time movement modeling (ctmm) framework to estimate dry season (26 ranges from 22 different individuals), wet season (12 ranges from 10 different individuals), and annual range sizes (8 individuals), and reported the 95%, 50% AKDE, and 95% Minimum Convex Polygon (MCP) range sizes. We assessed how landscape characteristics influenced range size based on a broad array of 48 landscape metrics characterizing aspects of vegetation, water, and human features and their juxtaposition in the study areas. To identify the most relevant landscape metrics and simplify our candidate set of informative metrics, we relied on exploratory factor analysis and Spearman’s rank correlation coefficient. Based on this analysis we adopted a final set of metrics into our regression analysis. In a multiple regression framework, we developed candidate models to explain the variation in AKDE dry season range sizes based on the previously identified, salient metrics of landscape composition.

**Results:**

Elephant dry season ranges were highly variable averaging 792.0 km^2^ and 184.2 km^2^ for the 95% and 50% AKDE home ranges, respectively. We found both the shape and spatial configuration of agriculture and natural vegetation patches within an individual elephant’s range play a significant role in determining the size of its range. We also found that elephants are moving more (larger energy expenditure) in ranges with higher percentages of agricultural area.

**Conclusion:**

Our results provide baseline information on elephant spatial requirements and the factors affecting them in Myanmar. This information is important for advancing future land use planning that takes into account space-use requirements for elephants. Failing to do so may further endanger already declining elephant populations in Myanmar and across the species’ range.

## Introduction

The ability to understand how range size and movement patterns of a species vary in changing landscapes is important for informing decision processes and landscape planning efforts by resource managers and conservation agencies [1, 2]. Information on space requirements across different levels of human presence on a landscape can guide planning efforts and ensure success of management objectives. The advent of GPS technology in wildlife telemetry has revolutionized how movement data are collected in the field of wildlife science [3, 4]. The ability to collect large volumes of location data with high temporal resolution allows robust inference on spatial requirements, including home range size, range shifts by season, and movement patterns within the home range. When paired with a powerful open-source technology, such as Google Earth Engine and R, understanding of the spatial context of the movement and space use patterns can be determined with relative ease. Such information allows scientists to address key conservation challenges, advancing ecological knowledge of a species and serving to answer applied questions [5–7].

To understand the drivers of an animal’s movements, it is critical to appropriately understand the landscape context influencing its movement decisions [8]. Traditionally, ecologists have used software, such as FRAGSTATS, to quantify landscape metrics [9] and address related ecological questions of interest [10]. However, new analytical approaches are providing ecologists with more flexibility and unified workflow within one programming environment such as R [11]. Easy extraction and quantification of landscape conditions using such platforms allow ecologists to carry out further analysis, such as data visualization, exploratory factor analysis, and generalized linear regression, to make inference on the ecological influence of landscape variables [6] with greater ease. Coupling such information with data on animal space use can allow deeper insight to how landscape characteristics shape space-use relationship, such as home range behavior.

The endangered Asian elephant (*Elephas maximus*) is particularly susceptible to habitat loss being the largest terrestrial mammal with large and heterogeneous habitat requirements [12–14]. The species is facing serious anthropogenic pressure across its geographic range [15–18]. Agricultural expansion is driving habitat fragmentation and loss, and is resulting in significant increase in human-elephant conflicts (often the killings of people and elephants). The combined effects of habitat loss and increased conflict represent a major threat to remaining elephant populations across Asia. This is exacerbated by the persistent threat of poaching to the survival of remaining elephant populations [16, 18, 19].

Myanmar, home to approximately 1,400 wild elephants [20], has the largest amount of remaining wildlands among the species range countries (37.86%) although the landscape is changing rapidly [16, 21]. The status of Myanmar’s elephants is unclear, but likely elephants are declining as they continue to face threats in the wild [21, 22]. Recent evidence of increased poaching is a serious concern [19]. At the same time, range loss, driven by rapid development across the country due to recent changes in the political system and an increased development focus [23], is thought to be the primary driver of elephant decline in the country. One study suggested that the geographic distribution of elephants in Myanmar declined by 5% (~ 15,000 km^2^) between 1992 and 2006 [22]. Even within a proposed national park in Myanmar, forest cover is declining [24]. There are only a few studies that have assessed the space use of wild Asian elephants [14, 25–27], and only one study assessing ranging behavior of wild elephants in Myanmar in relation to seed dispersal [28] to our knowledge. Therefore, it is crucial to obtain information relating space use and ranging behavior of elephants to their landscape context in the country.

Developing tools for assessing elephant space use and ranging requirements becomes even more critical with continued habitat loss. As human populations continue to increase, human encroachment into the remaining “wildlands” within the elephant’s range countries is likely to accelerate. This encroachment will inevitably lead to increased human-elephant encounters and conflicts. Additionally, increased fragmentation due to habitat loss could result in increased range size as elephants are forced to move further to meet the resource requirements [26, 29]. Elephants are likely to change their ranging pattern (area used and movement rates) in response to fragmentation and resource availability, and this is particularly relevant in the dry season in Southeast Asia when the configuration of resources varies across the landscape and availability of high-value food items (and resulting conflict) increases during the harvesting period [13, 28].

We looked at the relationship between animal space use and landscape context, by deriving metrics describing shape and configuration of land cover types (agriculture, water, and natural vegetation) within individual ranges. Our two main objectives of this study were to (1) quantify dry season range sizes in Myanmar and assess how ranging behavior during the dry season varies based on different configurations of available agriculture and natural vegetation (including testing for range size thresholds relative to percentage of agriculture); and (2) evaluate how percentage of agriculture within dry core range (50% AKDE range) of elephants relates to their daily distance traveled. In addition, we examined wet season ranging and annual ranging behaviors where data allowed.

## Methods

### Study areas

**Fig. 1:**
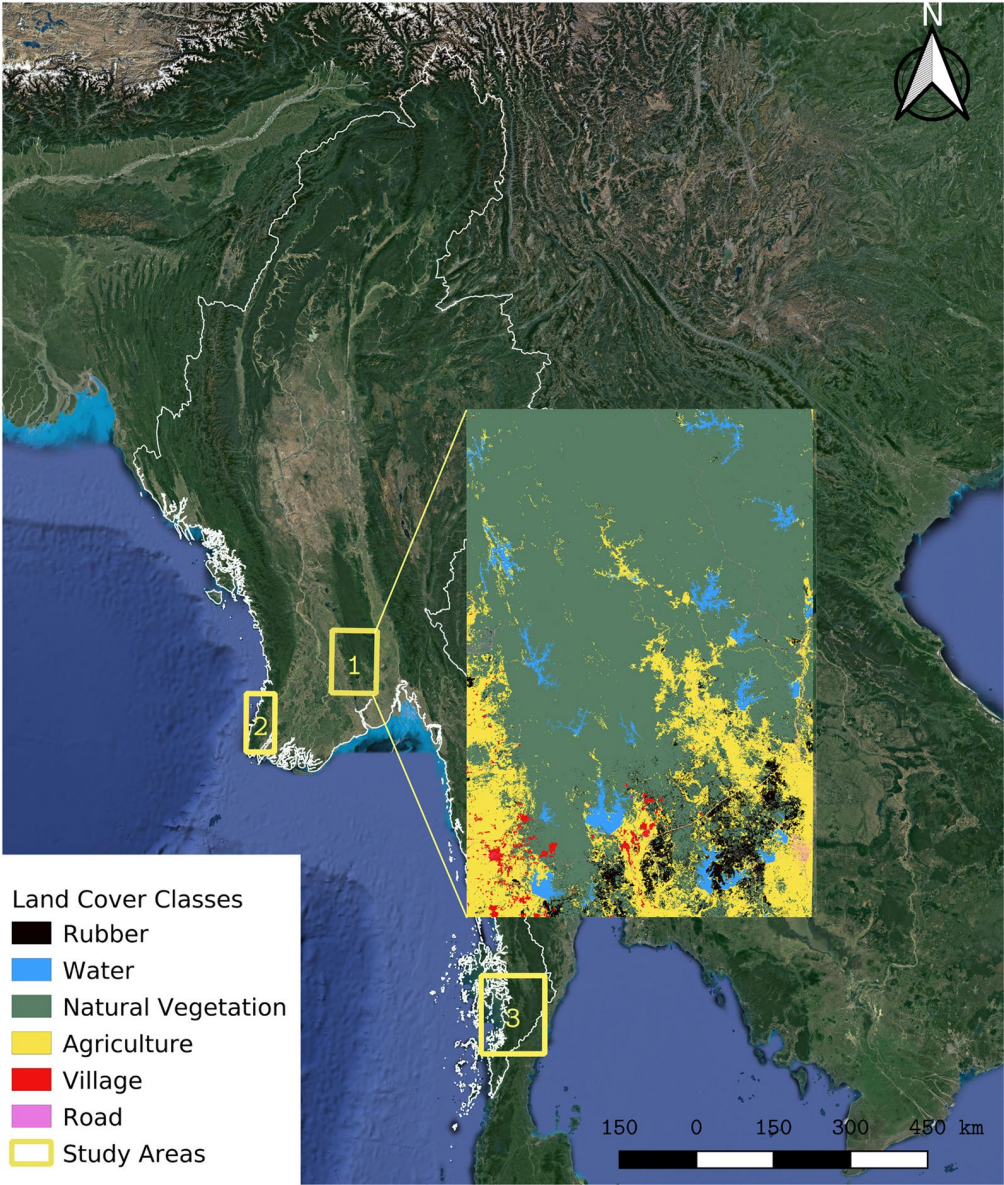
The location of the three study areas in Myanmar: Site 1 located in the foothills of Bago Yoma Mountain Range, site 2 located within the Ayeyarwaddy Delta region, and site 3 which is part of Dawna Tanintharyi Mountain Range. The insert shows the land cover map for site 1 from which various landscape metrics were derived for analysis of range conditions

Our study was conducted in three areas of conservation interest in central, western, and southern Myanmar (Fig. 1). Site 1 (Latitude: 17.1013–18.1960, Longitude: 95.7043–96.4787) is located in the central part of Myanmar in the foothills of Bago Yoma mountain ranges. Historical unsustainable teak extraction in this site created a highly disturbed forest mosaic that is increasingly being invaded by other human land uses, including the construction of hydroelectric reservoirs, settlements, as well as commercial teak, sugarcane, rice, and rubber plantations. Site 2 (Latitude: 16.0554–17.0842, Longitude: 94.1860–94.6838) is a mountainous area along the west coast of the Ayeyarwaddy state that stretches from north to south creating an elongated forest with hard boundaries on east and west. Rice plantations dominate the matrix between forest patches in this site, where rubber and peppercorn agricultural use is also prevalent. Site 3 (Latitude: 10.7141–12.0981, Longitude: 98.3356–99.4626) is part of the larger Dawna Tanintharyi Landscape which extends from mountain ridges along the border with Thailand to the coastal plain. Land use at site 3 is primarily composed of oil palm and betel nut plantations, surrounded by lowland deciduous forests. Threats of human encroachment, road development, and agricultural expansion into the remaining forest are rising in the area.

The study areas are strongly seasonal, with rainfall records demonstrating the extended dry season occurs between early December and late March, and the wet season between early June and mid-September [30]. During the dry season, human-elephant conflicts (HECs) peak in relation to the harvest of rice, sugarcane, and other agricultural products [29, 31]. Rainfall is significantly higher at site 3, resulting in markedly different forest composition. Forests at site 3 are predominantly lowland evergreen forests, while at site 1 and 2, they are mostly mixed deciduous forests with strong seasonal leaf-fall patterns.

### Elephant capture for GPS collaring

All capture and animal sedation were performed by veterinarians from Myanmar Timber Enterprise (MTE). MTE is the Myanmar government agency responsible for the management of logging elephants, and their staff have extensive experience in veterinary care of captive and wild Asian elephants, including sedation. Individuals were independently captured during the collaring period, and no collared elephants were found in the same social unit. All capture and handling procedures followed or exceeded the guidelines provided by the American Society of Mammalogists [32]. Elephants were immobilized using Etrophine and Xylaxine for sedation and Naltrexone for reversal. The immobilization and collaring process took approximately 30 min per individual on average and was carried out early in the morning or late in the afternoon when air temperature was relatively lower (< 35 °C). All the collars are set to record a GPS fix every hour. Due to high collar failure and poaching soon after collar deployments [19], telemetry datasets were often patchy and covered relatively short periods. Consequently, we only included individuals with (1) > 60 days of tracking data and/or (2) that had an established range (based on a semi-variogram analysis of range stability described below). To assess whether animals established ranges during the tracking periods, we used methods described by [33] in their continuous-time movement modeling package (ctmm) in R. When the semi-variogram function for the relocation data of an elephant approached an asymptote, we classified that dataset as capturing an established range [33], which occurred within 60 days each season for the elephants in this study.

For dry season range analysis, we analyzed data from eight individuals from site 1 (4 females: 4 males), six from the site 2 (1 female: 5 males), and eight from site 3 (2 females: 6 males)—totaling 22 different individuals. We performed data analysis on data collected from December 2016 through March 2020. Therefore, our analysis covers four dry seasons. There were four individuals whose tracking periods covered two dry seasons. To avoid problems with pseudo-replication when developing our regression model set, we excluded the year with fewer data points for each of these four individuals such that each only supplied one season to the analysis.

For the wet season ranges estimation, we utilized data from five individuals from site 1 (1 female: 4 males), three from the site 2 (3 males), and two from site 3 (2 males). There were two individuals who had data spanning two wet seasons, allowing estimation of 12 wet-season ranges. Because of the relatively small sample size, we did not run a regression analysis on wet season data.

For annual home range estimation, we included individuals with > 365 days tracked, which amounted to 8 individuals: five individuals from site 1 (1 female: 4 males), one from the site 2 (1 male), and two from site 3 (2 males).

### Range estimation

We employed a ctmm framework [33] to estimate seasonal (dry and wet) and annual range sizes among individuals. We compared the fit to our data of independent and identically distributed (IID), Ornstein–Uhlenbeck (OU), and Ornstein–Uhlenbeck Foraging (OUF) movement models using an autocorrelation estimation method. We picked the best fitting model and applied it to fit the autocorrelated density estimator (AKDE) function to estimate range size. We calculated 95% and 50% AKDE percentile level ranges for all individuals. We assumed 50% AKDE level as core areas within the respective ranges where animals spent 50 percent of their time. To enable comparison with other studies, we calculated and presented 95 percentile Minimum Convex Polygon (MCP) ranges.

### Predictor variables and candidate models

To assess which landscape conditions were related to dry season range size, we applied gamma regression models with estimated AKDE range sizes as a response variable based on the assumption that our response variable can only be a non-zero positive number. Our covariate dataset of landscape properties was developed by classifying Landsat 8 imageries to develop land cover maps for each of our study areas (Chan et al. unpublished data). We used the ‘landscapemetrics’ package in R to derive different measures for characterizing landscape metrics from our land cover map [11]. To describe the landscape of each individual range, we calculated several different shape, area, edge, and aggregation metrics for water, agriculture, and natural vegetation classes (Table 1). In addition, we quantified landscape-level metrics, including Shannon’s diversity index, relative patch richness, and relative patch density (Table 1). We computed 48 landscape metrics in total.

**Table 1:** Description of landscape metrics used in this study [11]

Abbreviations	Full name	Metric type	Description
frac_mn_*	Mean fractal dimension index	Shape	Fractal dimension based on the patch perimeter and patch area: value (x) approaches 1 if all patches are squared and 2 if all patches are irregular
frac_sd_*	Standard deviation of fractal dimension index	Shape	Standard deviation of the fractal dimension index, where x = 0 if the fractal dimension index is identical for all patches and increases without limit as the variation of the fractal dimension indices increases
para_mn_*	Mean perimeter to area ratio	Shape	A patch complexity metric that approaches 0 if the perimeter-to-area ratio for each patch approaches 0 (i.e. the form approaches a rather small square) and increases without limit, as perimeter-to-area ratio increases (patches become more complex)
para_cv_*	Coefficient of variation of perimeter to area ratio	Shape	Coefficient of variation of perimeter-area ratio where x = 0 if the perimeter-area ratio is identical for all patches and increases without limit as the variation of the perimeter-area ratio increases
para_sd_*	Standard deviation of perimeter to area ratio	Shape	Standard deviation of perimeter-area ratio where x = 0 if perimeter-to-area ratio is identical for all patches and increases without limit as the variation of the perimeter-area ratio increases. This is scale dependent
area_cv_*	Coefficient of variation of patch area	Area and edge	Summarizes variation in patch area where x = 0 if all the patches are identical in size and increases without limit as the variation of patch area increases in the landscape
area_mn_*	Mean patch area	Area and edge	This is the simplest metrics—mean patch area of a given class. If all patches are small, x = 0 and increases without limit as the patch areas increases
pland_*	Percentage of landscape	Area and edge	Characterizes the composition of the landscape as percentage of class *. When the proportional class area is decreasing, the value approaches 0. The metric is equal to 100 when only one patch is present on the landscape
pd_*	Patch density	Aggregation	Describes the fragmentation of the class as patch density where x approaches 0 as the proportional class area decreases. It is equal to 100 when only one patch is present. It is standardized to 100 hectares area
dcore_mn_*	Mean number of disjunct core area	Core area	This counts the disjunct core areas, whereby a core area is a patch within the patch containing only core cells. If ncore = 0 for all patches, x = 0 and increases without limit as the number of disjunct core area increases
dcad_*	Disjunct core area density	Core area	This is the number of disjunct core areas per ha relative to the total area. When no patch of class * contains a disjunct core area, x = 0, and increases without limit as disjunct core areas become more present (i.e. patches becoming larger and less complex)
ed_*	Edge density	Area and edge	Describes the configuration of the landscape as the sum of all edges of class * in relation to the landscape area. If only one patch is present, x = 0, and increases without limit as the landscape becomes more patchy
lsi_*	Landscape shape index	Aggregation	Metric based on actual edges and minimum hypothetical edges. When only one squared patch is present or all patches are maximally aggregated, x = 1, and increases without limit as the length of the actual edges increases (i.e. the patches become less compact)

To simplify these 48 metrics for our regression analysis, we relied on exploratory factor analysis with oblique minimal rotation of principal factor axes to reduce the data dimensionality. This approach relaxes the assumption of normality [34] and allowed us to identify the variables that best characterized variations in our landscapes (Table 1). Specifically, we included the highest positive and negative loading variables from the first five principal factor axes to reduce the metrics to the primary explanatory variables while explaining sufficient variance in the data. Afterwards, we compared single variable models among metrics belonging to the same land cover class. We kept the variables if the AIC corrected for small sample size (AICc) score was within 8 of the top model and excluded variables that did not meet the criteria in our candidate model set. AICc is the metric used to rank the models in your candidate model set in such a way that the most parsimonious model will have the lowest AICc value among the model set. This allowed us to eliminate variables with relatively low explanatory power. We also assessed the Spearman’s rank correlation coefficients between all the variables before including them in the final candidate model sets (all the variables included in the model set were less than 0.6).

From the retained variables (Table 1), we then developed different biologically meaningful combinations of agriculture and natural vegetation indices in the model set for both the 95% and 50% AKDE level for dry season ranges. We included a model with a quadratic term for percentage of agriculture to determine whether we could assess the threshold relationship between agriculture and range size. We also assessed the effect of sex, site, and year by adding these covariates to our best performing model and ranked the models using AICc for both 95% and 50% AKDE top models. We investigated these effects further in 50% AKDE range sizes analysis by dropping uninformative parameters in our model sets based on model weights and parameter estimates and presented the most parsimonious and biologically meaningful model since the effect of site/region came out stronger in our model set [35].

In addition to our range size models, we developed a candidate model set to assess the correlation between landscape metrics and average daily distance moved by the elephants. We calculated average daily distance moved by calculating the sum of hourly distance moved (straight-line distance between the two consecutive points) and dividing by the total number of days tracked for the particular individuals. We did not include days where fix success rate was below 80% in our daily distance traveled calculation. For this particular candidate model set, we tested several hypotheses using the most informative variables from the 50% AKDE dry season analysis. We tested whether sex, site/region, and/or two agriculture metrics (percentage of agriculture presence and perimeter-area ratio of agriculture patches within the range) influenced average daily distance moved by elephants by fitting gamma regression model as described above. We set female and study site 2 as a reference category for sex and region categorical predictor variables, respectively, in the model.

We used AICc to rank models in the candidate model set [36]. We selected the model with the lowest AICc as the best/top model in respective candidate model set. To account for variation in range sizes driven by sampling differences, we included the number of days tracked as an additional variable in the top model. We retained the number of days tracked variable if it was included in a model within 2 AICc scores of the top model. All variables were standardized to a mean of 0 and a standard deviation of 1 before fitting the model for easier interpretation of the results and standardize the effect size of all covariates. All analyses were conducted in R version 3.6.3 using ‘ggplot2’ (version 3.3.0), ‘dplyr’ (version 0.8.5), ‘ctmm’ (version 0.5.9), ‘landscapemetrics’ (version 1.4.3), and ‘AICcmodavg’ (version 2.2.2) [37–41].

## Results

### Determinants of seasonal home range size

Home range size estimates varied across seasons and individuals (Table 2). Elephant dry season ranges were highly variable averaging 792 km^2^ (± 867.6 km^2^; range from 38.4 km^2^ to 3,166.4 km^2^) for the 95% AKDE ranges while the 50% AKDE range sizes averaging 184.2 km^2^ (± 201.5 km^2^; range 7.4 to 728.5 km^2^). Despite more limited sample sizes (n_wet_ = 12, n_annual_ = 8), analysis of wet season range indicated the average 95% AKDE ranges was 1,520 km^2^ (range 43.5–5362.2 km^2^), and the average AKDE 50% ranges was 356.1 km^2^ (range 12.8–1277.5 km^2^). Considering only full annual ranges, the average range covered 1093.1 km^2^ (range 89.6–3057.4 km^2^) and 252.9 km^2^ (range 20.3–777.2 km^2^) for 95% and 50% AKDE home ranges respectively. We did not find differences in range sizes between males and females, probably because of the overall large variation in range size (average and standard deviation of female 50% AKDE = 153 km^2^ ± 221 km^2^; average and standard deviation of male 50% range 196 km^2^ ± 199 km^2^). The variation between the sites was greater for the 50% AKDE range size (including site as a covariate improved the explanatory power), but the effect of the site did not add much to the explanatory power of the best performing model of our 95% AKDE range analysis (Site 1: ${\overline{\text{x}}}$  = 750.3 ± 892.5 km^2^; Site 2: ${\overline{\text{x}}}$ = 947.4 ± 945.6 km^2^; Site 3: ${\overline{\text{x}}}$ = 713.3 ± 865.9 km^2^).

**Table 2:** Estimated 95 and 50 percentile AKDE ranges, and 95 percentile minimum convex polygon area in squared kilometers for dry season, wet season and annual range

ID	Year	Site	Dry MCP 95%	Wet MCP 95%	Annual MCP 95%	Dry AKDE 95%	Dry AKDE 50%	Wet AKDE 95%	Wet AKDE 50%	Annual AKDE 95%	Annual AKDE 50%
17,104	2017	Site 3	244.8	243.1	302.7	703.6	185.2	697.1	175.0	513.9	142.1
17,104	2018	Site 3	NA	164.7	NA	NA	NA	795.5	166.4	NA	NA
17,105	2017	Site 3	109.3	100.9	284.1	975.5	213.1	454.5	92.6	600.0	152.5
19,970	2016	Site 1	105.9	200.2	340.2	248.3	63.5	412.0	86.8	529.3	98.5
19,971	2016	Site 1	201.7	240.9	1153.0	1509	355.7	3662.2	875.2	2780.4	635.8
22,912	2016	Site 1	229.2	113.4	575.1	223.8	51.8	3977.2	914.0	775.6	121.5
22,912	2017	Site 1	91.3	128.7	NA	502.2	89.6	230.9	56.9	NA	NA
IRI2016-3121	2019	Site 3	57.3	NA	NA	65.7	11.7	NA	NA	NA	NA
IRI2016-3122	2019	Site 3	50.8	NA	NA	72.7	17.1	NA	NA	NA	NA
IRI2016-3123	2019	Site 3	184.3	NA	NA	2543.7	584.6	NA	NA	NA	NA
IRI2016-3124	2019	Site 3	292.1	NA	NA	1170.5	257.9	NA	NA	NA	NA
IRI2016-3125	2019	Site 3	89.2	NA	NA	146.5	35.7	NA	NA	NA	NA
ST2010-2594	2017	Site 3	14.3	NA	NA	28.4	7.4	NA	NA	NA	NA
ST2010-2707	2017	Site 2	132.4	141.1	NA	1545.4	381.6	2059.2	477.7	NA	NA
ST2010-2710	2017	Site 2	191.2	NA	NA	2790.9	644.7	NA	NA	NA	NA
ST2010-2710-REDEPLOY	2018	Site 1	150.6	NA	NA	591.9	143.8	NA	NA	NA	NA
ST2010-2711	2017	Site 3	139.9	NA	NA	530.6	132.0	NA	NA	NA	NA
ST2010-2713	2017	Site 1	61.7	NA	NA	388.5	87.2	NA	NA	NA	NA
ST2010-2714-REDEPLOY	2018	Site 1	180.9	NA	NA	433.7	85.6	NA	NA	NA	NA
ST2010-2716	2018	Site 1	74.1	27.9	65.8	171.3	40.7	43.5	12.8	89.6	20.3
ST2010-2716	2019	Site 1	77.4	NA	NA	151.6	29.5	43.5	NA	NA	NA
ST2010-2853	2018	Site 2	82.3	NA	NA	638.6	148	NA	NA	NA	NA
ST2010-2854	2018	Site 2	31.3	26.8	NA	64	15.9	54.9	14.3	NA	NA
ST2010-2855	2018	Site 2	113.6	160.3	262.4	857.6	214.4	486.1	124.2	398.3	74.9
ST2010-2855	2019	Site 2	78.9	NA	NA	204.6	42.4	NA	NA	NA	NA
ST2010-2856	2018	Site 1	188.4	239.6	678.8	867	222.2	5362.2	1277.5	3057.4	777.2
ST2010-2856	2019	Site 1	184.8	NA	NA	3166	728.5	NA	NA	NA	NA
	Average		129.14	170.27	492.97	791.9	184.2	1519.5	356.1	1093.1	252.8

**Fig. 2:**
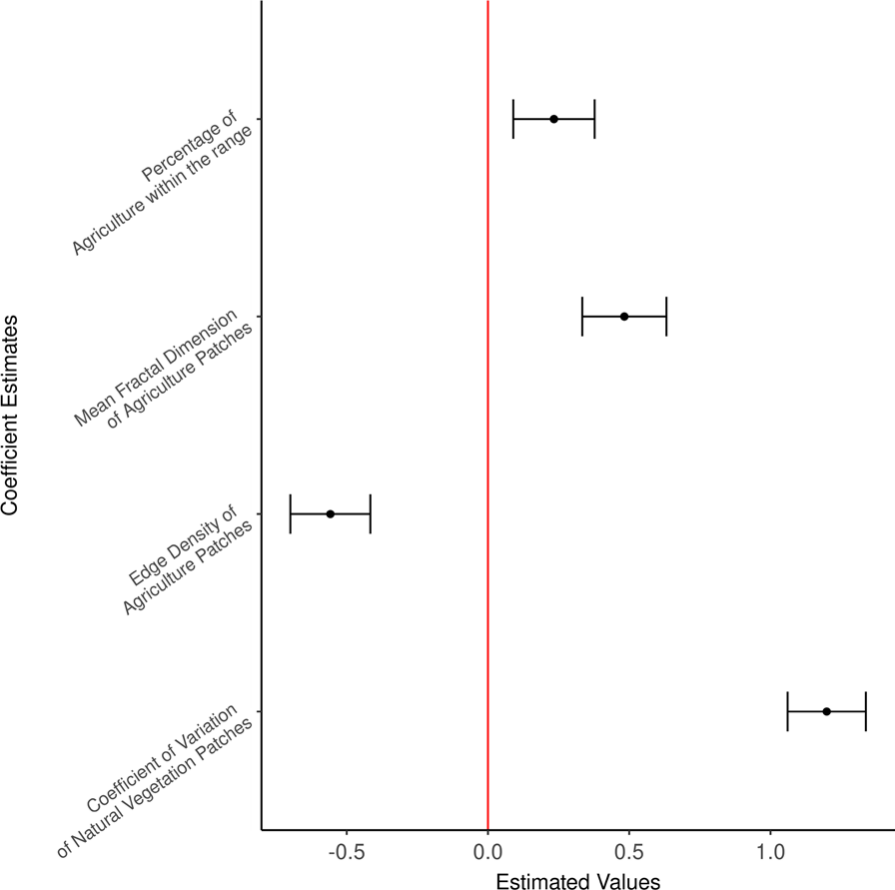
Estimated coefficient values from the top model of dry season 95% AKDE range showing landscape metrics describing the patterns of agriculture and variability in natural vegetation cover were the important independent variables in explaining variation in range size

**Fig. 3:**
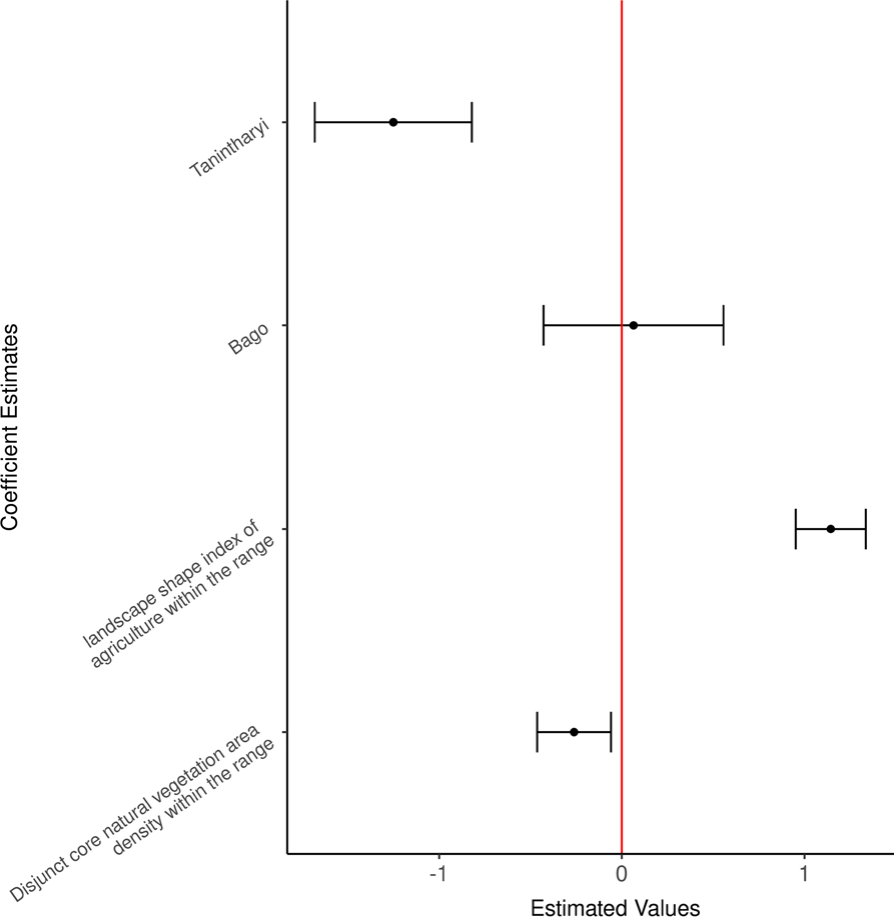
Estimated coefficient values from the top model of dry season 50% AKDE range showing landscape shape index for agriculture and several metrics representing natural vegetative constitution were the covariates explaining variation in range size. Site 2 (Ayeyarwaddy Delta region) is set as the reference site when fitting the model

The variation in 95% AKDE level dry season ranges was best explained by metrics characterizing agricultural land use rather than those of natural areas (Table 3); whereas metrics describing landscape configuration of natural vegetation classes explained the difference in 50% AKDE range sizes (core range area) (Table 4). The top models contained four statistically significant variables with three agriculture and one natural vegetation metrics in the top model for 95% AKDE ranges; and one metric for each agriculture and natural vegetation and site variable for 50% AKDE ranges (Figs. 2, 3). To assess the effects of sex, site, year on ranging behavior, we ran a secondary model including these variables in the top model of 95% AKDE range sizes. These variables did not add any significant explanatory power to our top model (δAICc is greater than 4).

**Table 3:** Candidate model set for 95% AKDE dry season range showing the performance of the top model relative to others in the model set

Model	Variables	AICc	K	dAICc	AICc weights
M_Ag_Nv_1	(Intercept) + pland_ag + frac_mn_ag + ed_ag + area_cv_natveg	284.93	5	0.00	0.89
M_Ag_Nv_2	(Intercept) + pland_ag + I(pland_ag^2) + frac_mn_ag + ed_ag + area_cv_natveg	289.18	6	4.26	0.11
M_Global	(Intercept) + area_cv_natveg + ed_ag + pland_ag + I(pland_ag^2) + dcore_mn_water + frac_mn_ag + para_mn_natveg + para_mn_water	299.78	9	14.85	0.00
M_Ag_Nv_3	(Intercept) + ed_ag + pland_ag + area_cv_natveg	306.08	4	21.16	0.00
M_Nv_W_2	(Intercept) + area_cv_natveg + para_mn_water	312.09	3	27.17	0.00
M_Nv_W_1	(Intercept) + area_cv_natveg + para_mn_water + dcore_mn_water	315.38	4	30.45	0.00
M_Ag_W_1	(Intercept) + pland_ag + frac_mn_ag + ed_ag + para_mn_water + dcore_mn_water	322.88	6	37.96	0.00
M_Water	(Intercept) + para_mn_water + dcore_mn_water	338.71	3	53.79	0.00
M_Null	(Intercept)	339.37	1	54.44	0.00
M_Ag_W_2	(Intercept) + pland_ag + frac_mn_ag + ed_ag + para_mn_water	343.15	5	58.23	0.00
M_Ag_1	(Intercept) + pland_ag + frac_mn_ag + ed_ag	346.12	4	61.20	0.00
M_Ag_2	(Intercept) + pland_ag + I(pland_ag^2) + frac_mn_ag + ed_ag	347.80	5	62.87	0.00

The top model is composed of three landscape metrics describing configuration and composition of agriculture and one regarding natural vegetation composition within the range

**Table 4:** Candidate model set for 50% AKDE dry season showing the top model carrying the majority of the model set weight (85.28%) composed of one metric describing the shape of the agriculture patches and three metrics describing shape and configuration of natural vegetation patches within the range

Model	Variables	AICc	K	dAICc	AICc weights
M_Ag_Nv_2	(Intercept) + lsi_ag + dcad_natveg + dcore_mn_natveg + para_mn_natveg	238.98	5	0	0.88
M_Global	(Intercept) + dcad_natveg + dcore_mn_natveg + para_mn_natveg + area_mn_natveg + lsi_ag	243.38	6	4.4	0.10
M_Ag_Nv_3	(Intercept) + lsi_ag + dcore_mn_natveg + dcad_natveg	247.04	4	8.06	0.02
M_Ag_Nv_1	(Intercept) + lsi_ag + dcore_mn_natveg	251.5	3	12.52	0
M_Ag	(Intercept) + lsi_ag	253.47	2	14.48	0
M_Nv_4	(Intercept) + dcad_natveg	273.91	2	34.92	0
M_Null	(Intercept)	275.17	1	36.19	0
M_Nv_3	(Intercept) + dcad_natveg + dcore_mn_natveg	276.32	3	37.33	0
M_Nv_2	(Intercept) + dcad_natveg + dcore_mn_natveg + para_mn_natveg	278.97	4	39.98	0
M_Nv_1	(Intercept) + dcad_natveg + dcore_mn_natveg + para_mn_natveg + area_mn_natveg	281.11	5	42.13	0

**Fig. 4:**
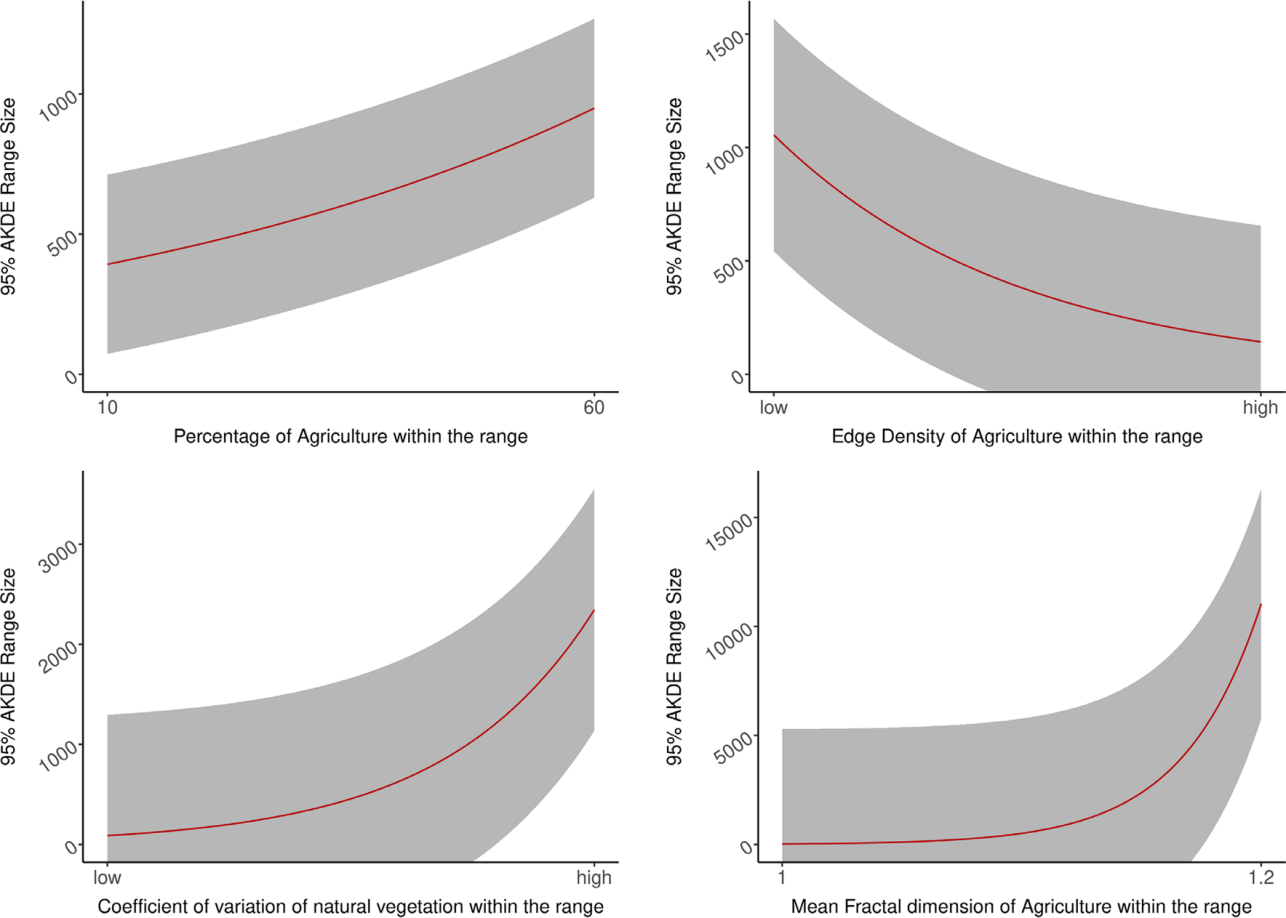
Functional relationship between the estimated regression coefficients of the top predictive landscape metrics and the dry season 95% AKDE range size. Predicted range size for elephants during the dry season increased as the landscape becomes more irregular and dominated with agriculture

The top model for 95% AKDE range included significant coefficient estimates for percentage of agriculture on the landscape, fractal dimension mean and edge density of agriculture, and the coefficient of variation of patch area for natural vegetation (Fig. 2 and Table 3). In general, elephants tend to have larger 95% AKDE range when the shape of agriculture patches were irregular (higher mean fractal dimension) and agriculture land use percentage on a landscape increased (Fig. 2). On the other hand, more patchy agriculture on a landscape (higher edge density) corresponded to smaller 95% AKDE range (Fig. 4). On average, one unit increase in the metric describing variation in natural vegetation patches (1 standard deviation from the mean) resulted in a 3.17 km^2^ increase in potential range size while holding the rest of the variables in the model at their mean value (Fig. 4). The likelihood ratio based r-squared for our top model was 0.9533.

**Fig. 5:**
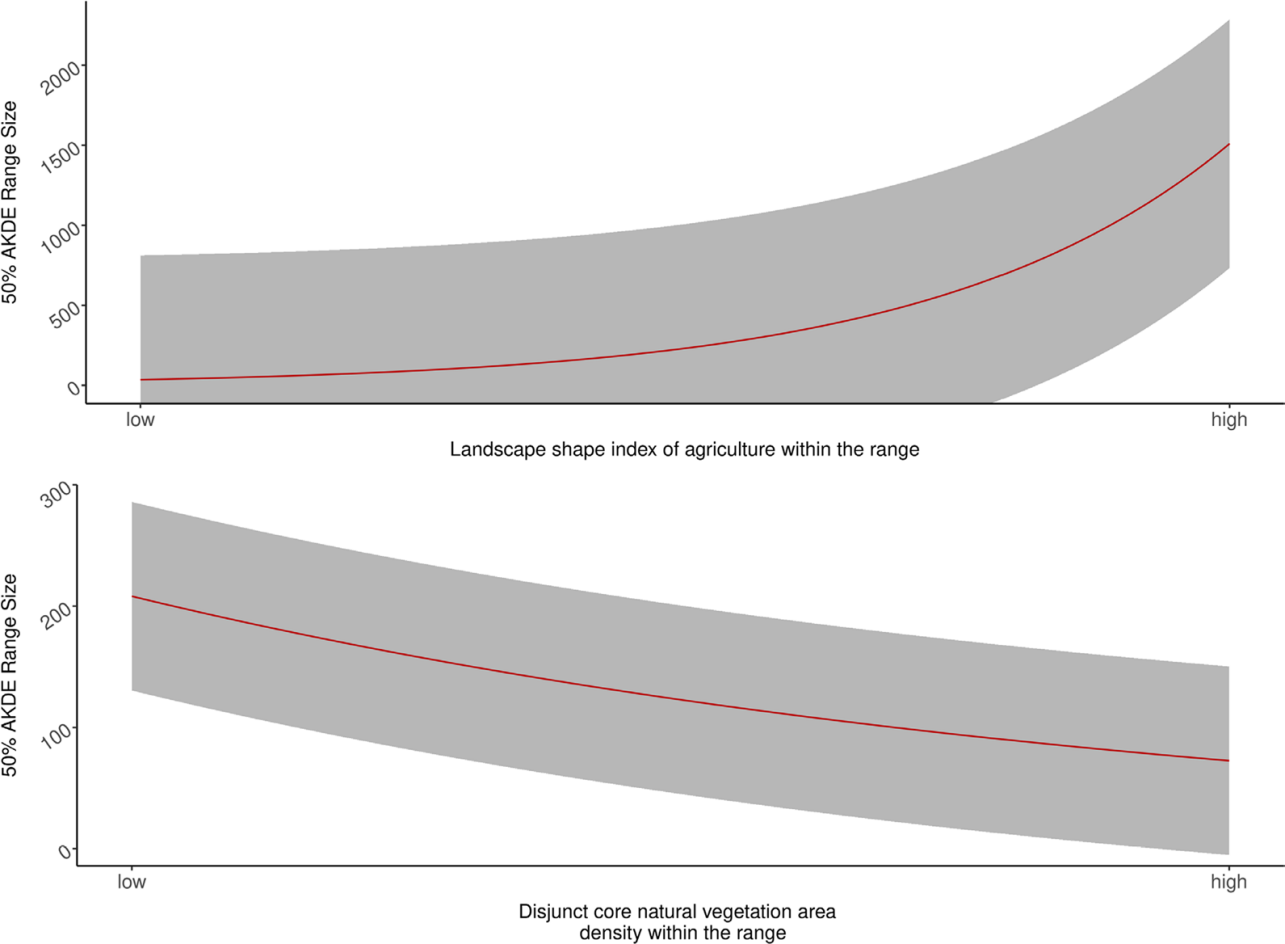
Functional relationship between the estimated regression coefficients of the top predictive landscape metrics of the dry season 50% AKDE range size. Predicted 50% AKDE range size for elephants during the dry season increased as the index of agriculture shape (i.e., agricultural boundary length) increased and decreased where more intact natural vegetation was found

The top model for 50% AKDE of the estimated dry season range included significant coefficient estimates for disjunct natural vegetation area density, landscape shape index of agriculture, and the site variable as a factor (reference site: site 2) (Fig. 3, Table 5). Smaller core range areas corresponded to more complex natural vegetation patches (i.e., increase in perimeter-area ratio, Fig. 5). In contrast, larger core range sizes corresponded with to less compact patches of agriculture (i.e. higher landscape shape index, Fig. 5). On average, an increase in 1 unit of landscape shape index score of agriculture (1 standard deviation from the mean) corresponded to an increase of 3.13 km^2^ in core range area. Estimated 50% AKDE ranges are smaller in Tanintharyi (Site 3) than in the reference site Ayeyarwaddy delta (Site 2) (Fig. 3). The likelihood ratio based R-squared for our top model is 0.929.

**Table 5:** Evaluating the effect of sex, site, and year on the differences in core range sizes on the best performing model of Table 4

Model	Variables	AICc	K	dAICc	AICc weights
M_Site_1	(Intercept) + lsi_ag + dcad_natveg + regionBago + regionTanintharyi	229.98	5	0.00	0.85
M_Sex_Site	(Intercept) + lsi_ag + dcad_natveg + Sexmale + regionBago + regionTanintharyi	234.16	6	4.18	0.11
M_Site_2	(Intercept) + lsi_ag + dcad_natveg + dcore_mn_natveg + para_mn_natveg + regionBago + regionTanintharyi	237.14	7	7.16	0.02
M_Sex	(Intercept) + lsi_ag + dcad_natveg + dcore_mn_natveg + para_mn_natveg + Sexmale	238.53	6	8.54	0.01
M_Ag_Nv_2	(Intercept) + lsi_ag + dcad_natveg + dcore_mn_natveg + para_mn_natveg	238.98	5	9.00	0.01
M_year	(Intercept) + lsi_ag + dcad_natveg + dcore_mn_natveg + para_mn_natveg + season2017_2018 + season2018_2019 + season2019_2020	251.77	8	21.79	0

### Daily travel distance

The average daily distance traveled by the elephants in the dry season was 3.9 km (range 1.3–7.3 km), with males moving 3.8 km (± 1.6 km; n = 19) and females 4.1 km (± 1.6 km; n = 7) per day. According to our top model, the average daily distance moved was 3.8 km at 13.9 percent agriculture within their home range (Table 6). Percentage of agriculture on a landscape (pland_ag) was the only covariate in our most parsimonious model (top model) to explain variation in average daily travel distance by elephants. An approximate increase of 15 percent in agriculture on the landscape resulted in an increase of 1.2 km in the daily distance traveled by the elephants. Study sites and sex of the individual were not included in the top model in our sample (Table 6).

**Table 6:** Candidate model set for average daily distance moved showing percentage of agriculture present within the 50% AKDE dry season range was the best variable examined at explaining the variation in mean average daily distance moved by the elephants during the dry season

Model	Variables	AICc	K	dAICc	AICc weights
M_3	(Intercept) + pland_ag	93.62	2	0.00	0.48
M_Null	(Intercept)	96.07	1	2.45	0.19
M_4	(Intercept) + pland_ag + Sexmale	96.20	3	2.58	0.16
M_1	(Intercept) + pland_ag + para_mn_ag	96.43	3	2.81	0.14
M_2	(Intercept) + pland_ag + site1 + site3	98.31	4	4.69	0.02
M_Global	(Intercept) + pland_ag + site1 + site3 + para_mn_ag	98.88	5	5.26	0.01

## Discussion

This is the first study to report different seasonal ranges (primarily dry season) of Asian elephants in Myanmar. The results show high variation in ranges sizes and demonstrated that some of this variation can be explained by differences in landscape metrics describing the relationship between natural vegetation and agriculture. We note there was greater variation in 95% AKDE range size within relative to between the three study sites across the country, suggesting strong variability between individual space use strategies. We also showed that elephants in Site 3 (Tanintharyi) had smaller core ranges. This could be the result of a high presence of palm oil plantations (plantations providing high-energy food in a relatively concentrated area) and the higher degree of fragmentation in natural vegetation throughout the southern landscape of Myanmar. Percentage of agriculture within the range positively correlated with average daily distance traveled by the elephants (i.e. elephants traveled further and faster in areas with higher percentage of agriculture). These findings demonstrate that elephants’ ranging behavior in Myanmar is influenced by different configurations of agriculture and natural vegetation on the landscape.

We identified percentage of agriculture, mean fractal dimension and edge density of agriculture (i.e., patchiness), and coefficient of variation in natural vegetation patches (i.e., differentiation between patch sizes) within an elephant’s range as the variables of importance in quantifying the level of fragmentation within an individual’s potential range (i.e., 95% AKDE range). Within the elephant’s core range (i.e., 50% AKDE range), we showed that landscape shape index for agriculture (i.e. the patch becomes less compact as the index increases) is the most important variable in explaining the variation in range sizes. We also showed that increase in agriculture resulting in loss of natural vegetation within elephants’ ranges corresponded to an increase in range sizes. We did not detect a relationship between range size thresholds relative to percentage of agriculture (our top model did not include the quadratic variable allowing such inference). However, further investigation on a larger data set would be valuable to determine the nature of this relationship. Sampling across a broader gradient of human agricultural use could provide more specific inference on this relationship, though it may be difficult to determine such thresholds if it is a gradual process and the number of elephants living near this theoretical threshold are small.

It is inevitable that Asian elephants will face increasing fragmentation and habitat loss due to agricultural expansion and urbanization across the range countries [16, 22, 42]. There is an evidence in the literature stating that the level of human footprint on a landscape can affect movement of animals [43]. Therefore, it is crucial to quantify the structure and magnitude of fragmentation within the species’ core range and understand the impact on animals’ movement behavior as a first step in any science-based management and conservation program. Previous research indicated that Asian elephants benefit from a mixture of natural vegetation and agriculture on a landscape [18, 22, 44]. Our results are in agreement with the existing literature on Asian elephant’s movement behavior in fragmented landscape, where elephants in more fragmented habitat are likely to move further (increased energy expenditure) to meet their survival and fitness requirements [26, 28, 29]. Increased movement may chiefly be a strategy whereby elephants reduce the inherent risk of being in close proximity to humans. For example, Evans et al. [45] found that Asian elephants on the island of Borneo moved faster in landscapes with increased human modification, presumably to avoid encounters with humans. However, elevated movement rates across human-modified landscapes may also be important to reduce poaching risks [46, 47]. This may be particularly true in Myanmar, where poaching for elephant skin has recently increased sharply across the agriculture-wildland interface [19].

Asian elephant range sizes are thought to be strongly determined by availability of water on a given landscape [13, 14]; however, the variables capturing water land cover class were not included in top models of neither 95% AKDE range nor core range sizes of elephants in our analysis for the dry season. This may indicate that water is not a limiting factor within these landscapes, possibly because elephants have already adjusted their range to meet their water requirement for the dry season or because water is relatively widely available. Alternatively, it is possible that the land cover map used in this study did not adequately capture all aspects of water availability on the landscape, or that the grain of our satellite imageries used to produce our land cover maps (30 × 30 m) was coarse to capture the seasonal variation of smaller water sources within our study sites.

To facilitate direct comparison between the results from this study and that of others, we reported MCP range sizes as well as AKDE. We found that dry season 95% AKDE range sizes ranged from 38.4 to 3,66.4 km^2^ in Myanmar, which is slightly larger compared to ranges reported in Sumatra, Indonesia using the same estimator (ranges from 275 to 5,179 km^2^) [27]. We estimated MCP annual ranges in Myanmar at 65.8 to 1152 km^2^ which shows more variation in range sizes than other studies using the same range estimation method—Sri Lanka: 51.2–179.2 km^2^ [14], Malaysia: 122–114 km^2^ [25], and India: 105–320 km^2^ [13]. All the compared studies were conducted either within protected areas or surrounded by protected areas; whereas, our study sites were primarily outside of the protected areas. In general, our study reported a lot more variations in range sizes since we included individuals from three different study sites across the country with different landscape configurations. This highlights further that ranging behavior of elephants are affected by land use types and their spatial configurations.

Our relatively small sample size of individuals and variable fix success across collars influenced our results to some extent. We relied on AKDE range estimates given they are relatively robust to differential sampling and fix success issues (average fix success rate ~ 75% during the wet season in this study). Notably, our AKDE analysis yielded larger range sizes for wet season than annual ranges, despite the annual range estimates including all data used to estimate the wet season range (in addition to data from the dry season) for some individuals. The larger AKDE wet season estimate was likely a result of the small sample size and temporally dispersed relocation points in the dataset that may result from dispersal behavior or a function of poor collar performance during the wet season causing more uncertainty in the estimates [48]. We calculated 95% minimum convex polygon and found annual ranges were larger than wet season ranges (Table 2). This is not intended as a comparison between the two estimators, but an exploration of the seasonal and total differences. Relatedly, we also estimated large ranges in the dry season for some individuals (particularly individual 1997) that had relatively lower fix success (~ 70 percent fix success rate during the dry season). While it is probable that fix success played a role in the estimates, these large ranges are likely biologically driven. For instance, individual 19,971 was a young male (15–25 y.o. estimated age) navigating the human-dominated landscape of Bago Yoma, and the large range size may be driven by physiological demands and reproductive strategies in the highly fragmented landscape [49].

Elephants continue to face habitat loss and fragmentation across their range due to development [16, 18]. This will in turn increase human-elephant encounters [29]. Although there are several ways to mitigate human-elephant conflicts particularly at the agriculture-wildland interface, such as electric fencing, bee fencing, and chili fencing, it is important to identify if we are mitigating the problem or simply moving it elsewhere [50, 51]. When deploying temporary or permanent fencing on a landscape, we are fragmenting the landscape, which can drive behavioral responses from the elephants. For instance, increased fragmentation in the study system is related to larger ranges. Mitigation approaches could cause the elephants to move more broadly, potentially spreading conflict areas across a broader area. Therefore, it is important to consider the impact of mitigation methods on elephants’ ranging behavior in a larger landscape scale although these mitigation methods could prove to be useful for given locations or when implemented strategically as part of a broader landscape planning effort. Our study provides a useful model to predict the degree to which ranging behavior of elephant in Myanmar could change based on changes in fragmentation on a landscape. For example, in Site 2 (Ayeyarwaddy Delta), an increase in 1 unit of landscape shape index score of agriculture (1 standard deviation from the mean, i.e., more patchy) corresponded to an increase of 3.13 km^2^ of core range area. Elephants may be able to persist in these heterogeneous agriculture-natural-vegetation landscape mosaics for the long term if human-elephant conflicts can be managed appropriately by targeting actions that keep human and elephant casualties low and reduce economic impacts on local farmers. To reach this goal, we must pay attention to changes in elephant space use in relation to land use development and human-elephant conflict mitigation actions to help ensure ecologically sustainable policy and decisions by mangers and conservationists. It is also important to ensure the remaining wildlands for elephants are protected, which will provide refuge habitat and could reduce the overall area use by elephants—range use increased with less natural area (Fig. 5). The degradation of remaining natural areas should be prevented at all cost to reduce negative interaction between human and elephants in the country.

Our models are evaluated for three study sites in Myanmar. We encourage managers and policy makers to re-evaluate our model using the same approach when extrapolating our results outside of the study areas. Increasing human footprint as a result of land use changes on a landscape will impact ranging and other movement behavior of the elephants [43, 45]. Therefore, information regarding the potential effect size of change on those behavior is taken into account during the decision making process to ensure elephants can exist in the area of concern.

## Conclusion

This study provides foundational information on the movement ecology and ranging behavior of Asian elephant in Myanmar. Although Myanmar has lower elephant number than countries such as Sri Lanka and India, it has large tracts of suitable habitat for Asian elephants, making it a key range country for the species [16]. Determining habitat requirements through studies of habitat selection and space use, can serve the country by providing managers and policy makers with concrete information on habitat requirements of this endangered species. This study provides such baseline information, while also providing insight to how landscape structures influence elephant space use. It also highlights the importance of assessing elephant use of areas outside of protected areas, which have been traditionally overlooked. Since it was predicted that 41.8% of the 256,518 km^2^ of the available habitat for Asian elephants will be lost by the end of century [52], we expect more fragmentation and land use changes within elephant’s core ranges which could potentially lead to larger ranging behavior increasing both the number of and distribution of human-elephant conflicts. We showed that increasing agriculture will lead to detrimental consequences on elephants, but determining the threshold will be difficult and could be the point of no return once a population gets there. Therefore, monitoring with the help of GPS tracking and high resolution satellite imageries, we can provide empirically sound information on how elephants are navigating in human-dominated landscapes and effectiveness of potential mitigation methods for HEC. We believe the species could benefit from us applying science-based management decisions for future land-use planning.

## Data Availability

Data will be available on request but can not be released publicly due to the endangered status of Asian elephants.

## References

[1] Morato RG, Stabach JA, Fleming CH, Calabrese JM, De Paula RC, Ferraz KMPM (2016). Space use and movement of a neotropical top predator: the endangered jaguar. PLOS ONE Public Libr Sci.

[2] Wadey J, Beyer HL, Saaban S, Othman N, Leimgruber P, Campos-Arceiz A (2018). Why did the elephant cross the road? The complex response of wild elephants to a major road in Peninsular Malaysia. Biol Conserv.

[3] Wall J, Wittemyer G, Klinkenberg B, Douglas-Hamilton I (2014). Novel opportunities for wildlife conservation and research with real-time monitoring. Ecol Appl.

[4] Kays R, Crofoot MC, Jetz W, Wikelski M (2015). Terrestrial animal tracking as an eye on life and planet. Science..

[5] Gorelick N, Hancher M, Dixon M, Ilyushchenko S, Thau D, Moore R (2017). Google Earth Engine Planetary-scale geospatial analysis for everyone. Remote Sens Environ..

[6] Seidel DP, Dougherty E, Carlson C, Getz WM (2018). Ecological metrics and methods for GPS movement data. Int J Geogr Inf Sci.

[7] Wittemyer G, Northrup JM, Bastille-Rousseau G (2019). Behavioural valuation of landscapes using movement data. Philos Trans R Soc B Biol Sci.

[8] Nathan R, Getz WM, Revilla E, Holyoak M, Kadmon R, Saltz D (2008). A movement ecology paradigm for unifying organismal movement research. Proc Natl Acad Sci.

[9] Lamine S, Petropoulos GP, Singh SK, Szabó S, Bachari NEI, Srivastava PK (2018). Quantifying land use/land cover spatio-temporal landscape pattern dynamics from Hyperion using SVMs classifier and FRAGSTATS®. Geocarto Int.

[10] Midha N, Mathur PK (2010). Assessment of forest fragmentation in the conservation priority Dudhwa landscape, India using FRAGSTATS computed class level metrics. J Indian Soc Remote Sens.

[11] Hesselbarth MHK, Sciaini M, With KA, Wiegand K, Nowosad J (2019). landscapemetrics: an open-source R tool to calculate landscape metrics. Ecography.

[12] Owen-Smith N (1988). Megaherbivores: the influence of very large body size on ecology.

[13] Sukumar R (1989). Ecology of the Asian elephant in southern India. I. Movement and habitat utilization patterns. J Trop Ecol.

[14] Fernando P, Wikrarnanayake ED, Janaka HK, Jayasinghe LKAA, Gunawardena M, Kotagama SW (2008). Ranging behavior of the Asian elephant in Sri Lanka. Mamm Biol.

[15] Santiapillai C, Jackson P (1990). The Asian elephant: an action plan for its conservation.

[16] Leimgruber P, Gagnon JB, Wemmer C, Kelly DS, Songer MA, Selig ER (2003). Fragmentation of Asia’s remaining wildlands: implications for Asian elephant conservation. Anim Conserv.

[17] Choudhury A, Lahiri Choudhury DK, Desai A, Duckworth J, Easa PS, Johnsingh AJ, et al. *Elephas maximus*. The IUCN Red List of Threatened Species. 2008.

[18] Calabrese A, Calabrese JM, Songer M, Wegmann M, Hedges S, Rose R (2017). Conservation status of Asian elephants: the influence of habitat and governance. Biodivers Conserv..

[19] Sampson C, McEvoy J, Oo ZM, Chit AM, Chan AN, Tonkyn D (2018). New elephant crisis in Asia—early warning signs from Myanmar. PLoS ONE Public Libr Sci.

[20] Leimgruber P, Wemmer C. National elephant symposium and workshop. Report to the USFWS and the Myanmar Forest Department. 2004.

[21] Leimgruber P, Oo Z, Aung M, Kelly D, Wemmer C, Senoir B (2011). Current status of Asian elephants in Myanmar. Gajah.

[22] Songer M, Aung M, Allendorf TD, Calabrese JM, Leimgruber P (2016). Drivers of change in Myanmar’s wild elephant distribution. Trop Conserv Sci.

[23] Prescott GW, Sutherland WJ, Aguirre D, Baird M, Bowman V, Brunner J (2017). Political transition and emergent forest-conservation issues in Myanmar. Conserv Biol.

[24] Connette GM, Oswald P, Thura MK, LaJeunesse Connette KJ, Grindley ME, Songer M (2017). Rapid forest clearing in a Myanmar proposed national park threatens two newly discovered species of geckos (Gekkonidae: Cyrtodactylus). PLoS ONE..

[25] Kumar MA, Mudappa D, Raman TRS (2010). Asian elephant, *Elephas maximus,* habitat use and ranging in fragmented rainforest and plantations in the Anamalai Hills, India. Trop Conserv Sci.

[26] Alfred R, Ahmad AH, Payne J, Williams C, Ambu LN, How PM (2012). Home range and ranging behaviour of bornean elephant (*Elephas maximus borneensis*) females. PLoS ONE..

[27] Moßbrucker AM, Fleming CH, Imron MA, Pudyatmoko S, Sumardi (2016). AKDEC home range size and habitat selection of Sumatran elephants. Wildlife Research..

[28] Campos-Arceiz A, Larrinaga AR, Weerasinghe UR, Takatsuki S, Pastorini J, Leimgruber P (2008). Behavior rather than diet mediates seasonal differences in seed dispersal by Asian elephants. Ecology.

[29] Fernando P, Wikramanayake E, Weerakoon D, Jayasinghe LKA, Gunawardene M, Janaka HK (2005). Perceptions and patterns of human-elephant conflict in old and new settlements in Sri Lanka: Insights for mitigation and management. Biodivers Conserv.

[30] Biswas S, Vadrevu KP, Lwin ZM, Lasko K, Justice CO (2015). Factors controlling vegetation fires in protected and non-protected areas of Myanmar. PLoS ONE.

[31] Sukumar R (2003). The living elephants.

[32] Sikes RS (2016). 2016 Guidelines of the American Society of Mammalogists for the use of wild mammals in research and education. J Mammal.

[33] Calabrese JM, Fleming CH, Gurarie E (2016). ctmm: an r package for analyzing animal relocation data as a continuous-time stochastic process. Methods Ecol Evol.

[34] Costello AB, Osborne JW (2005). Best practices in exploratory factor analysis: Four recommendations for getting the most from your analysis. Pract Assess Res Eval.

[35] Arnold TW (2010). Uninformative parameters and model selection using Akaike’s information criterion. J Wildl Manag.

[36] Burnham KP, Anderson DR (2002). Model selection and multimodel inference.

[37] Wickham H (2016). ggplot2: elegant graphics for data analysis.

[38] Mazerolle MJ. AICcmodavg: model selection and multimodel inference based on (Q)AIC(c). R package version 2.2-2. 2019.

[39] Wickham H, François R, Henry L, Müller K. dplyr: a grammar of data manipulation. R package version 0.8.5. 2020.

[40] Fleming CH, Calabrese JM. ctmm: continuous-time movement modeling. R package version 0.5.9. 2020.

[41] R Core Team. R: a language and environment for statistical computing. Vienna, Austria; 2020.

[42] Sodhi NS, Koh LP, Brook BW, Ng PKL (2004). Southeast Asian biodiversity: an impending disaster. Trends Ecol Evol.

[43] Tucker MA, Böhning-Gaese K, Fagan WF, Fryxell JM, Van Moorter B, Alberts SC (2018). Moving in the Anthropocene: global reductions in terrestrial mammalian movements. Science.

[44] Fernando P, Leimgruber P. Asian elephants and seasonally dry forests. Smithsonian Institution Scholarly Press; 2011. p. 151–63.

[45] Evans LJ, Goossens B, Davies AB, Reynolds G, Asner GP (2020). Natural and anthropogenic drivers of Bornean elephant movement strategies. Glob Ecol Conserv.

[46] Sukumar R (1990). Ecology of the Asian elephant in southern India. II. Feeding habits and crop raiding patterns. J Trop Ecol..

[47] Webber CE, Sereivathana T, Maltby MP, Lee PC (2011). Elephant crop-raiding and human-elephant conflict in Cambodia: crop selection and seasonal timings of raids. Oryx.

[48] Fleming CH, Calabrese JM (2017). A new kernel density estimator for accurate home-range and species-range area estimation. Methods Ecol Evol.

[49] Taylor LA, Vollrath F, Lambert B, Lunn D, Douglas-Hamilton I, Wittemyer G (2020). Movement reveals reproductive tactics in male elephants. J Anim Ecol.

[50] Barua M, Bhagwat SA, Jadhav S (2013). The hidden dimensions of human-wildlife conflict: health impacts, opportunity and transaction costs. Biol Cons.

[51] Shaffer LJ, Khadka KK, Van Den Hoek J, Naithani KJ (2019). Human-elephant conflict: a review of current management strategies and future directions. Front Ecol Evol.

[52] Kanagaraj R, Araujo MB, Barman R, Davidar P, De R, Digal DK (2019). Predicting range shifts of Asian elephants under global change. Divers Distrib.

